# Efficacy and safety of anlotinib in combination with ¹³¹I therapy in the treatment of distant metastatic differentiated thyroid cancer: a single-arm, phase II study

**DOI:** 10.3389/fonc.2025.1622676

**Published:** 2025-07-14

**Authors:** Yan Li, Jianjing Liu, Qian Su, Xueyao Liu, Zhen Yang, Zhao Yang, Jie Fu, Yan Zhang, Lina Tong, Fang Yang, Dong Dai

**Affiliations:** ^1^ Department of Nuclear Medicine, Tianjin Cancer Hospital Airport Hospital, National Clinical Research Center for Cancer, Tianjin, China; ^2^ Department of Molecular Imaging and Nuclear Medicine, Tianjin Medical University Cancer Institute and Hospital, National Clinical Research Center for Cancer, Key Laboratory of Cancer Prevention and Therapy, Tianjin’s Clinical Research Center for China, Tianjin, China

**Keywords:** anlotinib, ^131^I therapy, distant metastatic differentiated thyroid cancer, efficacy, safety

## Abstract

**Background:**

Radioactive iodine (RAI) is the standard treatment for distant metastatic differentiated thyroid cancer (dmDTC). However, many patients fail to achieve satisfactory effects and prognosis. Anlotinib is a highly effective antiangiogenic tyrosine kinase inhibitor (TKI) that has shown promising efficacy in RAIR-DTC patients. This study evaluated the efficacy and safety of anlotinib in combination with ^131^I in dmDTC.

**Methods:**

This single-arm, phase II study was prospectively registered on the Chinese Clinical Trial Registry (ChiCTR2500095313). The key eligible criteria included patients with dmDTC who had at least one measurable metastatic lesion capable of iodine uptake and were planned to receive RAI therapy. Previous treatment with TKI was not permitted. Patients underwent a whole-body iodine scan (Rx-WBS) following iodine administration on days 3-5. When confirmed iodine uptake in metastatic lesions, anlotinib would be given at 12 mg (QD, 2 weeks on/1 week off, Q3W) initially. One combination treatment cycle consisted of 12 weeks of anlotinib and 1 dose of iodine-131. The primary endpoints were the objective response rate (ORR) and changes in thyroglobulin (Tg) levels. The secondary endpoints included disease control rate (DCR), progression-free survival (PFS), and safety.

**Results:**

From October 2022 to September 2024, 20 patients (4 males and 16 females) with distant metastatic DTC were enrolled. All patients who had completed at least one cycle of combined treatment were eligible for data analysis. The median follow-up was 13.7 months. 11 patients achieved partial response (PR), 8 patients achieved stable disease (SD), and 1 patient had progressive disease (PD). ORR and DCR were 55.0% [95% Confidence Interval (CI): 31.5%-76.9%] and 94.7% (95% CI: 75.1%-99.9%) respectively. Median PFS was not reached. All patients achieved a biochemical response according to protocol-defined criteria, defined as a ≥25% decrease in Tg levels. Grade 3 or higher treatment-related adverse events (TRAEs) were observed in 10 (50%; most common hypertension) patients. Dose reductions of anlotinib were required in 10 (50%) patients due to AEs, and no patient discontinued treatment because of AEs. No serious adverse events (SAEs) or deaths were reported.

**Conclusions:**

This study demonstrates the promising efficacy and safety of combining the TKI with ^131^I therapy, suggesting that anlotinib may be a viable option for dmDTC.

**Clinical Trial Registration:**

https://www.chictr.org.cn/showproj.html?proj=226033, identifier ChiCTR2500095313.

## 1 Introduction

Radioactive iodine (RAI) remains the cornerstone treatment for distant metastatic differentiated thyroid cancer (dmDTC) (1). However, a significant proportion of patients either do not achieve a satisfactory therapeutic effect or eventually develop RAI-refractory DTC (RAIR-DTC), leading to a poorer prognosis (2). This challenge highlights the urgent need for novel therapeutic strategies to overcome RAI resistance and improve outcomes in dmDTC.

Anlotinib is a novel, multi-targeted tyrosine kinase inhibitor (TKI) that primarily functions by inhibiting angiogenesis and suppressing tumor proliferation through its actions on various targets, including VEGFR, FGFR, PDGFR, and c-Kit (3, 4). While anlotinib has demonstrated promising efficacy as a monotherapy in advanced RAIR-DTC (5), a growing body of evidence suggests a synergistic potential when TKIs are combined with RAI therapy (6).

The rationale for combining TKIs like anlotinib with RAI stems from their complementary mechanisms of action. Preclinical and clinical studies have indicated that TKIs can potentially re-differentiate RAIR-DTC cells, thereby restoring or enhancing their iodine-uptaking capability (7). This “re-sensitization” to iodine, often achieved by inhibiting pathways involved in dedifferentiation (such as MAPK or PI3K/Akt pathways, which TKIs can modulate), allows for the re-application of effective RAI therapy in previously refractory cases. Furthermore, anlotinib’s direct anti-angiogenic and anti-tumor proliferative effects can independently contribute to tumor control and potentially reduce tumor burden, creating a more favorable microenvironment for RAI uptake and efficacy (8, 9). The combination aims to leverage these distinct yet synergistic mechanisms: anlotinib addresses tumor growth and potentially enhances iodine avidity, while ¹³¹I delivers targeted cytotoxic radiation to iodine-avid cells (10, 11). This dual approach is hypothesized to provide a more comprehensive and potent anti-tumor response compared to either monotherapy alone, particularly in patients with dmDTC.

Recent efforts have begun to explore the potential benefits of TKI and RAI combination strategies. For example, a phase II clinical trial by Song et al. investigated sequential use of anlotinib followed by RAI in patients with advanced DTC, demonstrating promising efficacy and safety (6). Additionally, the ongoing prospective trial NCT04988248 is evaluating lenvatinib in combination with RAI in iodine-avid metastatic thyroid cancer (12). These studies highlight a growing interest in integrated approaches, further supporting the rationale for our investigation.

Building upon this compelling preclinical and early clinical evidence, this study aimed to further evaluate the efficacy and safety of anlotinib in combination with ¹³¹I therapy in patients with distant metastatic differentiated thyroid cancer, with the goal of identifying a novel and effective treatment paradigm for this challenging patient population.

## 2 Materials and methods

### 2.1 Study design

This was a single-arm, exploratory phase II trial designed to evaluate the preliminary efficacy and safety of anlotinib combined with radioiodine-131 (¹³¹I) in dmDTC patients. The rationale for combining anlotinib and ¹³¹I in this study was based on both biological plausibility and unmet clinical need. Anlotinib, a novel multi-target TKI, has been approved in China for the treatment of RAIR-DTC, yet its potential to enhance or restore RAI uptake in iodine-avid metastatic lesions remains underexplored. Unlike traditional studies that focus solely on TKI monotherapy in RAIR-DTC, this trial was specifically designed to evaluate whether the sequential combination of anlotinib and RAI could offer synergistic clinical benefits in patients with measurable, iodine-avid metastatic lesions.

In this study, we strictly required evidence of iodine avidity before initiating anlotinib therapy, thereby ensuring that each enrolled lesion had the biological potential to respond to RAI. The treatment protocol involving a 12-week course of anlotinib per cycle was determined based on prior pharmacokinetic and clinical tolerability data from the ALTER01032 study. In addition, our dose-adjustment strategy for anlotinib followed the manufacturer’s guidelines and clinical best practices, allowing sequential reductions (12 → 10 → 8 mg) based on adverse event severity and patient tolerance. All efficacy assessments followed Response Evaluation Criteria In Solid Tumors (RECIST) 1.1, and biochemical response was evaluated using Tg level reductions as a surrogate for residual tumor activity.

The sample size of 20 patients was not based on formal power calculations but was determined according to feasibility considerations and in alignment with similar early-phase studies in this population. The results of this trial are intended to inform the design of future large-scale randomized studies. The study adhered to Good Clinical Practice (GCP) guidelines and the ethical principles of the 2013 Declaration of Helsinki. It was approved by the Institutional Review Board (IRB) of Tianjin Cancer Hospital Airport Hospital (approval JS-2022-0047), which provided ongoing ethical oversight. All study procedures were conducted after obtaining written approval. The study is registered on the Chinese Clinical Trial Registry (ChiCTR, Identifier: ChiCTR2500095313). It can be accessed at: https://www.chictr.org.cn/showproj.html?proj=226033. Written informed consent was obtained from all participants before study initiation, confirming their voluntary participation and understanding of the study objectives, procedures, and potential risks and benefits.

### 2.2 Patients

Patients aged 18–70 years who had histologically or radiologically confirmed DTC with distant metastasis following total or subtotal thyroidectomy, with at least one measurable metastatic lesion [according to RECIST version 1.1] capable of iodine uptake and were planned to RAI therapy, and Eastern Cooperative Oncology Group performance status (ECOG PS) of 0 – 1, and adequate bone marrow, renal, hepatic, and cardiac function. Previous treatments with VEGFR TKIs, such as anlotinib, vandetanib, cabozantinib, lenvatinib, sorafenib, and RET inhibitors like pralsetinib, and selpercatinib, were not permitted. Patients who have received other localized antitumor therapy within the last 3 months, or who are scheduled to undergo systemic antitumor therapy external irradiation, or other interventions during the period of dosing in this study will not be allowed to enroll, including cytotoxic therapies, immunotherapies. Patients with factors that may affect the administration of oral medications (e.g., inability to swallow), severe and/or uncontrolled diseases, participated in other anti-tumor drug clinical trials within 3 months, or are currently enrolled in other clinical trials were not included in this study.

### 2.3 Study treatment

RAI therapy was administered using a fixed-dose protocol: 200 mCi (7.4 GBq) for pulmonary metastases and 250 mCi (9.25 GBq) for bone metastases (It should be noted that all enrolled patients had concomitant pulmonary and/or bone metastases). Patients underwent a whole-body iodine scan (Rx-WBS) following iodine administration on day 3-5. After confirmation of iodine uptake in metastatic lesions, anlotinib therapy was initiated. Patients initially received anlotinib at 12 mg (QD, 2 weeks on/1 week off, 3 weeks for 1 cycle). Each combined treatment cycle comprised 1 dose iodine-131 and a 12-week course of anlotinib treatment until disease progression, intolerability, non-iodine-avid metastatic lesions, or withdrawal from the study. Anlotinib may be administered at reduced doses of 10 mg/day and 8 mg/day sequentially if the patient experiences an intolerable adverse event. If more than 2 dose levels need to be reduced, treatment would be terminated.

### 2.4 Study endpoints

The primary endpoints were the ORR [defined as the number of patients achieving an overall best response of complete response (CR) or partial response (PR) divided by the total number of patients]) and biochemical response (BRR defined as a decreased Tg level ≥ 25%). The secondary endpoints included disease control rate (DCR), median progression-free survival (mPFS), and safety. Adverse events (AEs) were evaluated according to National Cancer Institute Common Terminology Criteria for Adverse Events version 5.0 (NCI-CTCAE 5.0). The efficacy assessment is performed every 12 weeks, according to RECIST 1.1 criteria.

### 2.5 Statistical analyses

All patients who had completed at least one combined treatment cycle (the intent-to-treat population) were included in the Full Analysis Set (FAS). The primary efficacy analyses would be based on FAS. The Safety Analysis Set (SAS) included all patients who had completed at least one combined treatment cycle. The ORR was calculated based on the best response and is presented with 2-sided 95% Clopper and Pearson confidence intervals (CIs). Patient demographic data and safety parameters were summarized using descriptive statistics. Median PFS and overall survival are presented with 2-sided 95% CIs and were calculated using the Kaplan-Meier method and plotted over time after therapy. Clinical statistical analyses were performed using SAS version 9.4.

## 3 Results

### 3.1 Patient characteristics

From October 2022 to September 2024, 27 patients were screened, and 7 were excluded because they did not meet the inclusion criteria. Finally, 20 patients (4 males and 16 females) with distant metastatic DTC were enrolled. All patients who had completed at least one cycle of combined treatment were eligible for data analysis. The baseline clinical characteristics of the patients are shown in **Table 1**. The median patient age was 59 years and had a baseline Eastern Cooperative Oncology Group performance status of 1 (60%). Forty percent of the patients have PTC,40%have FTC, and the remaining 20% have mixed cell carcinoma. The most common sites of distant metastases were bone (75%) and lung (60%). At the time of data cutoff (January 14, 2025), the median duration of follow-up was 13.7 months. Twelve patients had exited the study group. Among them, 7 patients stopped iodine treatment due to non-iodine-avid metastatic lesions, and 5 of these patients continued with anlotinib monotherapy. Four patients stopped iodine treatment due to personal preference, and 3 of these patients continued with anlotinib monotherapy. One patient received other treatments due to new lesions. Eight patients continued on treatment.

**Table 1:** Baseline characteristics of the study population (N=20).

Baseline characteristics	Value
Age, median (range)	59 (45-72)
Sex, n (%)	
Male	4 (20.0)
Female	16 (80.0)
ECOG PS, n (%)	
0	8 (40.0)
1	12 (60.0)
Clinical stage, n (%)	
II	8 (40.0)
IVB	12 (60.0)
Histologic subtypes, n (%)	
Papillary	8 (40.0)
Follicular	8 (40.0)
Mixed	4 (20.0)
Metastases site, n (%)	
Bone	15 (75.0)
Lung	12 (60.0)
Adrenal	2 (10.0)
Others	2 (10.0)
Number of metastases, n (%)	
1	14 (70.0)
2	6 (30.0)
History of surgery, n (%)	20 (100.0)
Previous RAI treatments, n (%)	
1	12 (60.0)
2	4 (20.0)
≥3	4 (20.0)
Prior RAI dose ≥600 mCi, n (%)	4 (20.0)

ECOG PS, Eastern Cooperative Oncology Group Performance Status; RAI, radioactive iodine.

### 3.2 Efficacy

**Figure 1:**
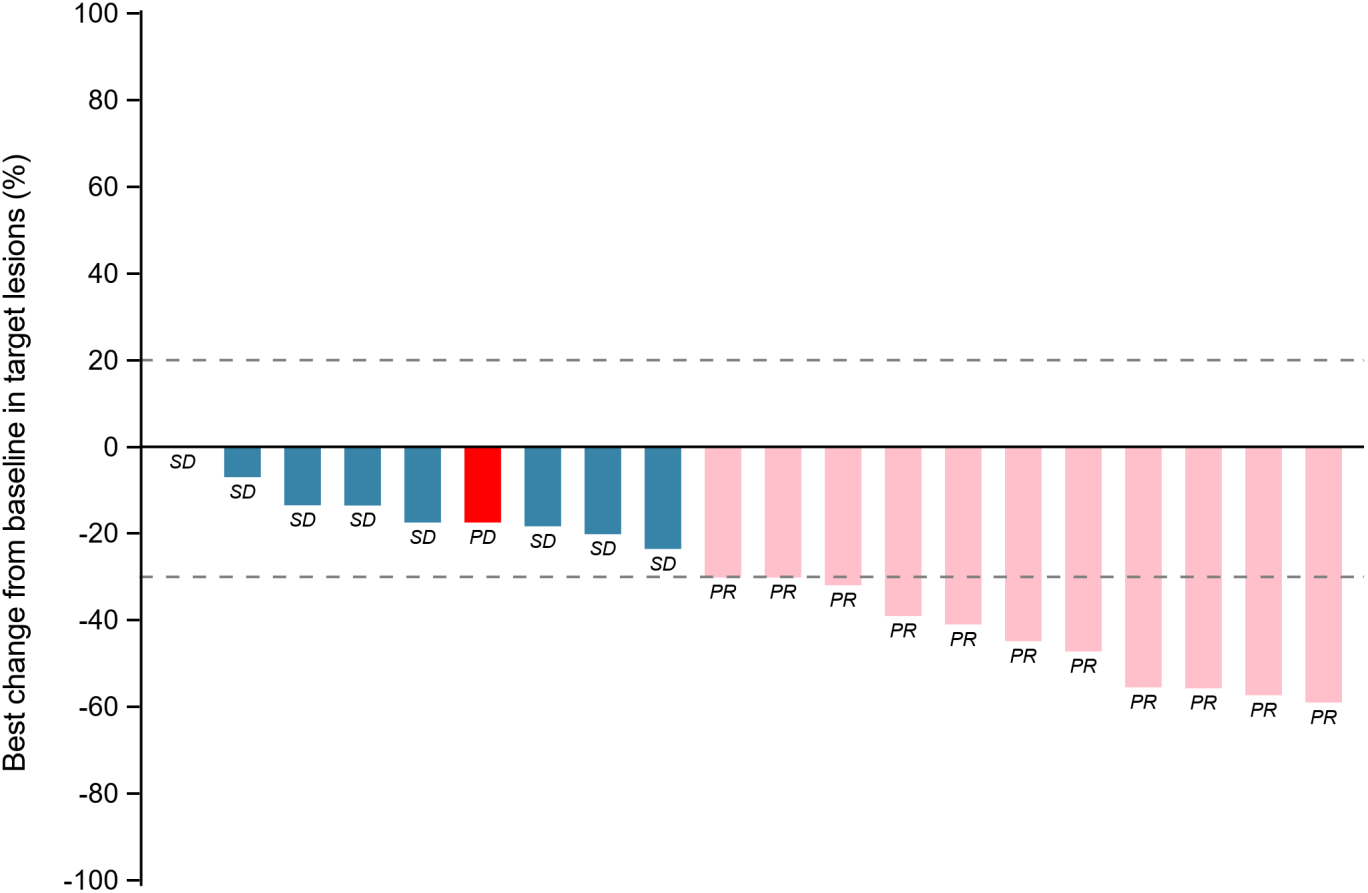
Tumor response assessment: maximum percentage change in target lesions from baseline by patient. Waterfall plot depicting the maximum percentage change in target lesion size from baseline for each patient. Bars are color-coded to indicate the best overall response. Blue: Stable disease (SD, change within −30% to +20%). Pink: Partial response (PR, ≥−30% decrease). Red: Progressive disease (PD, ≥+20% increase or new lesions). PR: partial response; SD: stable disease; PD: progressive disease.

**Figure 2:**
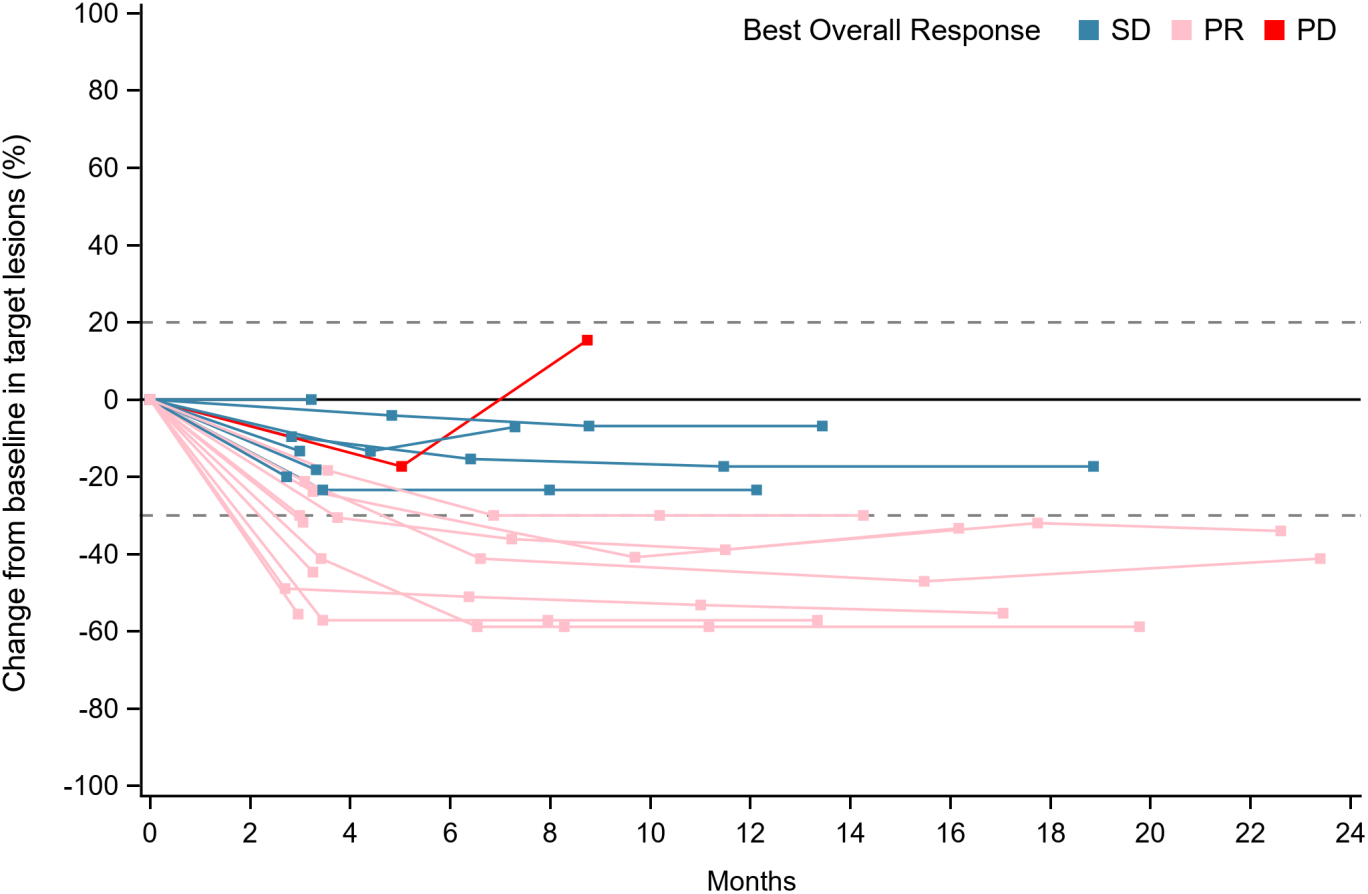
Longitudinal evaluation of tumor response categories over time. Swimmer’s plot showing individual patient responses (SD, PR, or PD) across the study duration (0–24 months). Each horizontal line represents a patient, with segments colored by response category. Blue: Stable disease (SD, change within −30% to +20%). Pink: Partial response (PR, ≥−30% decrease). Red: Progressive disease (PD, ≥+20% increase or new lesions). PR, partial response; SD, stable disease; PD, progressive disease.

**Figure 3:**
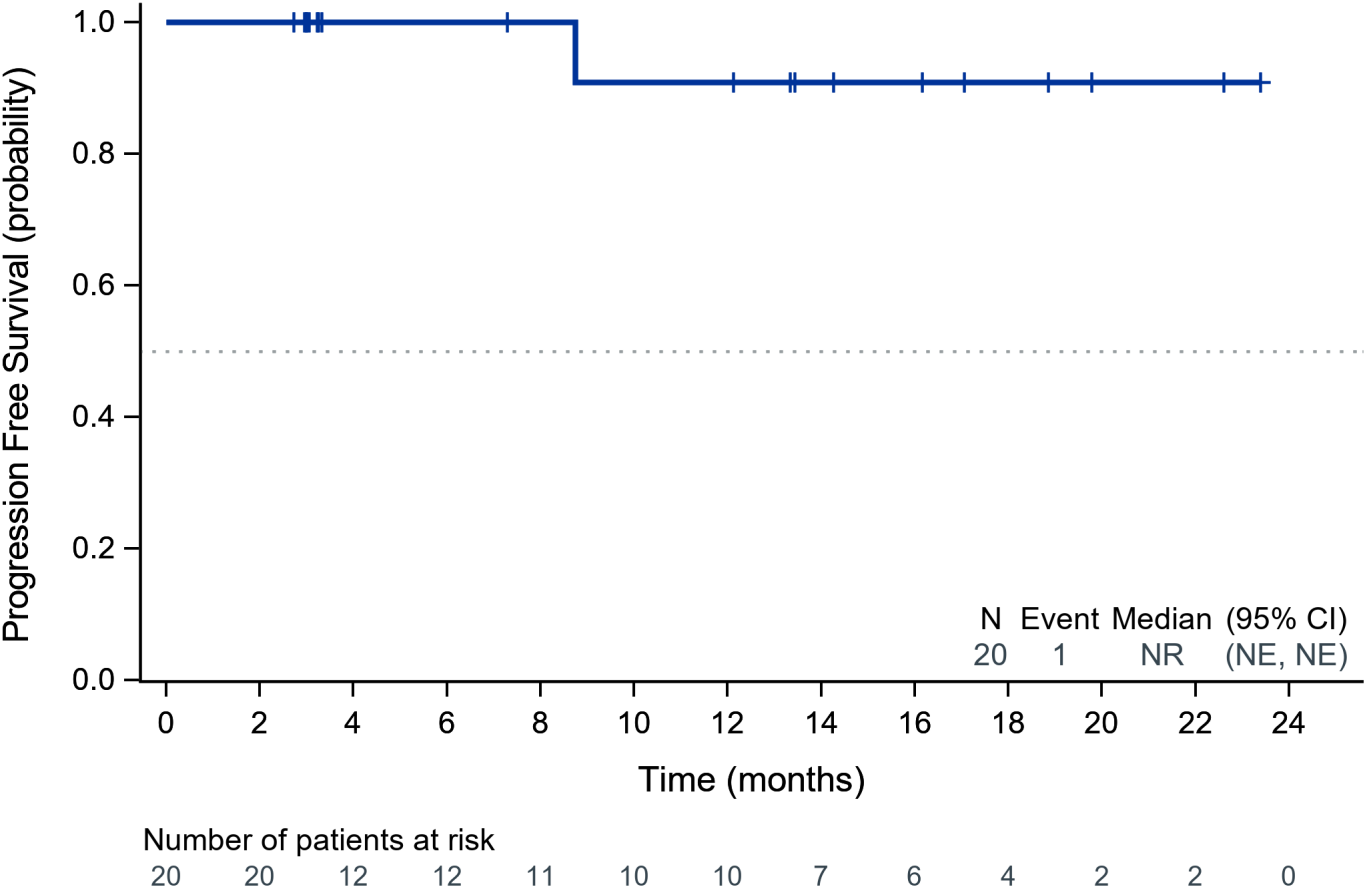
Kaplan-Meier analysis of progression-free survival in the study cohort. Kaplan-Meier curve illustrating PFS probability over time (months). The dashed line indicates the median PFS (95% CI). Numbers below the graph represent patients at risk at each timepoint. PFS, progression-free survival; CI, confidence interval.

**Figure 4:**
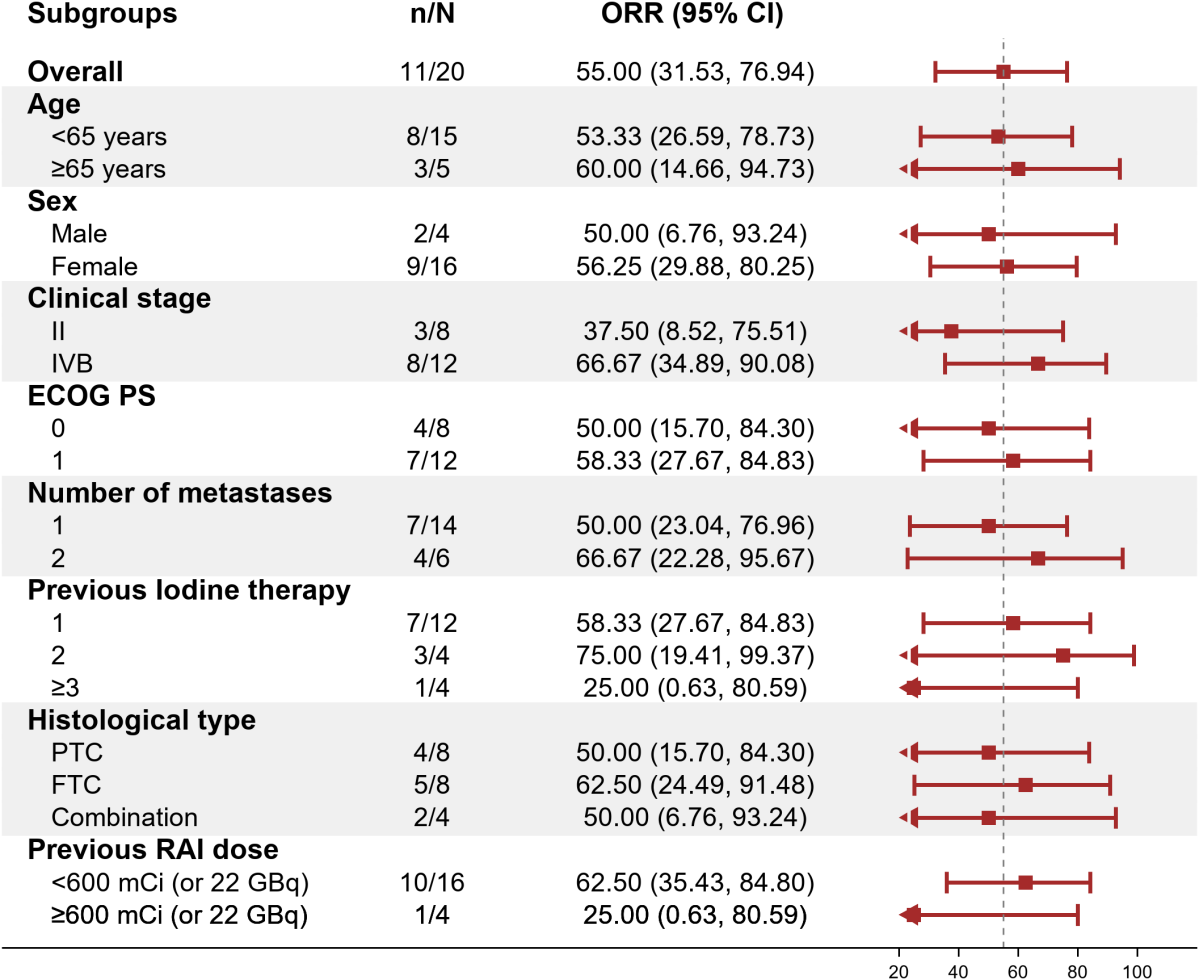
Objective response rate (ORR) across subgroups. Forest plot displaying ORR (95% CI) for prespecified subgroups (e.g., age, sex, clinical stage). The dashed vertical line marks the overall ORR (55%). ORR, objective response rate; CI, confidence interval; ECOG PS, Eastern Cooperative Oncology Group Performance Status; PTC, papillary thyroid carcinoma; FTC, follicular thyroid carcinoma; RAI, radioactive iodine.

Within the ITT population, none of the cases achieved a complete response. Eleven patients achieved PR, 8 achieved SD and 1 had PD because of new pulmonary lesions. ORR and DCR were 55.0% (95%CI: 31.5%-76.9%) and 94.7% (95%CI: 75.1%-99.9%) respectively. A waterfall plot of each efficacy-evaluable patient’s maximum percentage tumor change in the sum of the greatest dimensions from baseline to postbaseline nadir is provided in **Figure 1**, and the changes in each evaluation are shown in **Figure 2**. Median PFS was not reached at the cut-off date (**Figure 3**). After completing a full treatment cycle, all patients were observed to have achieved biochemical response, defined as a decrease in Tg levels of ≥25%. Eighteen patients (90%) experienced a Tg decrease of more than 50%. ORR for subgroups is shown in **Figure 4**.

### 3.3 Safety

All patients experienced at least 1 treatment-related adverse events (TRAEs), and the most common TRAE was hypertension, followed by hand-foot syndrome (**Tables 2**, **3**). Grade 3 or higher TRAEs were observed in 10 (50%) patients. Dose reductions of anlotinib were necessitated in 10 (50%) patients due to AEs, and no patient discontinued treatment because of AEs. No serious adverse events (SAEs) or deaths were reported.

**Table 2:** Incidence of treatment-related adverse events (TRAEs) (N=20).

TRAEs	All Grade, n (%)	Grade 1-2, n (%)	Grade 3-4, n (%)
Hypertension	16 (80.0)	10 (50.0)	6 (30.0)
Hand-foot syndrome	10 (50.0)	8 (40.0)	2 (10.0)
Hyperlipidemia	5 (25.0)	5 (25.0)	0
Fatigue	4 (20.0)	4 (20.0)	0
Decreased appetite	3 (15.0)	2 (10.0)	1 (5.0)
Diarrhea	3 (15.0)	3 (15.0)	0
Proteinuria	2 (10.0)	2 (10.0)	0
Gum pain	2 (10.0)	2 (10.0)	0
Thrombocytopenia	2 (10.0)	2 (10.0)	0
Oral mucositis	2 (10.0)	1 (5.0)	1 (5.0)
Angular cheilitis	1 (5.0)	1 (5.0)	0
Oropharyngeal pain	1 (5.0)	1 (5.0)	0
Lower limb edema	1 (5.0)	1 (5.0)	0

TRAEs, treatment-related adverse events.

**Table 3:** Summary of treatment-related adverse events (N=20).

Category	n (%)
Any TRAE	20 (100.0)
Grade ≥3 TRAE	10 (50.0)
Leading to dose reduction	10 (50.0)
Leading to treatment interruption	0
Leading to death	0
SAE	0

TRAE, treatment-related adverse event; SAE, serious adverse event.

## 4 Discussion

The prognosis of DTC markedly worsens with distant metastases, with 10-year survival rates decreasing from over 95% for localized disease to approximately 50% for metastatic cases (13). The lungs and bones are the most common metastatic sites, with bone metastases associated with poorer outcomes compared to pulmonary involvement (2, 14, 15). According to the 2015 American Thyroid Association (ATA) guidelines, RAI therapy is recommended for iodine-avid metastatic lesions and can be repeated when objective clinical benefits are demonstrated (1). However, durable complete remission remains rare—only 6.8% of metastatic DTC patients achieve disease-free status following RAI (16). Thies et al. (17) reported that patients requiring more than 22.2 GBq of ¹³¹I to achieve remission had comparable long-term outcomes to those who failed to achieve remission, highlighting the limitations of RAI monotherapy.

Approximately one-third of patients with distant metastases eventually lose their ability to concentrate RAI, progressing to RAIR-DTC (18). In this context, combining RAI with targeted therapies has gained increasing attention. Our study aimed to evaluate the efficacy and safety of combining anlotinib with ¹³¹I in patients with iodine-avid advanced DTC.

To contextualize our findings, we compared them with previous clinical trials involving anlotinib and other TKIs. The pivotal phase III ALTER0103 trial demonstrated that anlotinib monotherapy achieved an ORR of 59.21% and a DCR of 97.37% in patients with RAIR-DTC (5). Although our study design differed—our cohort included iodine-avid patients, and we utilized combination therapy rather than monotherapy—the observed ORR of 55% and DCR of 94.7% support a potential synergistic effect between anlotinib and RAI, particularly in patients who still retain iodine avidity. Importantly, our study was not designed as a direct comparator to anlotinib monotherapy, and further randomized controlled trials are needed to confirm these observations.

Lenvatinib and sorafenib, two multi-targeted TKIs, have been approved for the treatment of RAIR-DTC and represent foundational systemic options in this setting (3, 19). Although we did not directly compare our combination regimen to these agents, our findings contribute to the expanding evidence base supporting the role of TKIs in advanced thyroid cancer, while also highlighting the feasibility and potential advantages of integrating traditional RAI therapy with modern targeted agents. The broad inhibitory profile of anlotinib—targeting VEGFR, FGFR, PDGFR, and c-Kit—may play a role in enhancing the efficacy of this combination.

The rationale for combining anlotinib with RAI stems from their complementary mechanisms of action. RAI efficacy depends on adequate sodium-iodide symporter (NIS) expression for tumor uptake. However, in advanced or metastatic DTC, NIS expression is often downregulated due to activation of the mitogen-activated protein kinase (MAPK) and phosphatidylinositol-3-kinase (PI3K)/protein kinase B (AKT) pathways (20, 21). Anlotinib has been shown not only to inhibit angiogenesis via VEGFR2/3 but also to indirectly modulate the MAPK signaling cascade (4, 5), potentially restoring NIS function and enhancing RAI uptake in partially dedifferentiated tumors.

Other targeted therapies, such as selumetinib and dabrafenib, have also demonstrated the ability to restore RAI sensitivity through MAPK inhibition in both preclinical and early-phase clinical studies (22–24). Our approach differed in that we applied anlotinib after confirming iodine avidity, rather than using it as a redifferentiation agent. This strategy reflects a pragmatic and safer clinical design that aims to potentiate RAI responsiveness while minimizing the risk of overtreatment and drug-related toxicities.

The results of the ASTRA trial, a randomized phase III study, did not demonstrate significant benefit from using selumetinib prior to RAI in high-risk DTC patients after surgery (CR rate: 40% vs. 38%, P = 0.82) (25). In contrast, our patient selection focused exclusively on those with iodine-avid lesions, potentially avoiding premature systemic therapy and preserving RAI efficacy. This approach may partly explain the higher ORR (55%) and biochemical response observed in our cohort.

Our findings are also consistent with prior phase II studies that explored the combination of TKIs and RAI. In one such study, 16 patients with locally advanced or metastatic DTC received anlotinib (12 mg QD, 2 weeks on/1 week off) for four cycles. At week 13, diagnostic ¹³¹I whole-body scans (Dx-WBS) identified eight patients with iodine-avid lesions, who then underwent ¹³¹I treatment. Among them, the ORR was 33.3% and DCR was 83.3%, with biochemical responses observed in all patients (6). Similarly, Shi et al. (26) reported significant tumor reduction and SUVmax decline in five patients treated with apatinib and RAI. Song et al. also reported an ORR of 33.3% and complete biochemical responses following sequential anlotinib and RAI therapy (6). Additionally, an ongoing prospective trial (NCT04988248) is expected to provide further insights into the synergistic potential of lenvatinib and RAI in iodine-avid metastatic DTC (12).

Our study further explored the impact of cumulative RAI dosage on treatment efficacy. Patients with cumulative doses >600 mCi exhibited a lower ORR (25%) compared to those with <600 mCi (62.5%), suggesting that prolonged RAI exposure may reduce treatment sensitivity. We retrospectively reviewed 35 patients from our center who continued RAI therapy after exceeding 600 mCi. Although most lesions remained iodine-avid, none achieved radiological complete response. Only 14% (5/35) achieved >50% decline in thyroglobulin (Tg) levels, highlighting the limited efficacy of continued RAI beyond a certain threshold (unpublished data). These findings underscore the need to identify optimal timing for transitioning from RAI to systemic therapy.

Importantly, the combination of anlotinib with RAI was well-tolerated, with no additional adverse events observed compared to monotherapies. This indicates the safety and clinical feasibility of the combined regimen for appropriately selected patients with advanced, iodine-avid DTC.

This study, however, has several limitations. First, the single-arm design of this study precludes causal inference regarding the efficacy of anlotinib in combination with ¹³¹I. In the absence of a control group, it is not possible to rule out the influence of natural disease evolution or other external variables. This limitation restricts the interpretation of treatment effect size and hinders direct comparison with standard therapies. Future randomized controlled trials are necessary to confirm these preliminary findings and establish the causal relationship between the intervention and clinical outcomes. Second, the relatively small sample size (n=20) restricts the statistical power of our findings and may limit the generalizability of the results. It also means that rare adverse events might not have been captured, and some observed effects might be subject to greater variability. Finally, we also acknowledge that the relatively short follow-up period limits our ability to assess long-term outcomes, such as overall survival (OS), and the long-term safety profile of this combination therapy. We have also clearly stated that these limitations underscore the need for larger, randomized, controlled Phase III trials with longer follow-up periods to validate our preliminary findings and provide more robust evidence for the anlotinib-¹³¹I combination.

## 5 Conclusion

This phase II study demonstrates promising efficacy and manageable toxicity of anlotinib combined with ¹³¹I in distant metastatic DTC. Randomized studies are essential to confirm these findings and explore more therapeutic options for patients with distant metastatic DTC.

## Data Availability

The raw data supporting the conclusions of this article will be made available by the authors, without undue reservation.
